# Multifactorial Determinants of Target and Novelty-Evoked P300 Amplitudes in Children of Addicted Parents 

**DOI:** 10.1371/journal.pone.0080087

**Published:** 2013-11-14

**Authors:** Anja S. Euser, Brittany E. Evans, Kirstin Greaves-Lord, Ben J. M. van de Wetering, Anja C. Huizink, Ingmar H. A. Franken

**Affiliations:** 1 Department of Psychology, Erasmus University Rotterdam, Rotterdam, The Netherlands; 2 Department of Child and Adolescent Psychiatry/Psychology, Erasmus Medical Center/Sophia Children’s Hospital, Rotterdam, The Netherlands; 3 Department of Developmental Psychology and the EMGO Institute for Health and Care, VU University Amsterdam, Amsterdam, The Netherlands; 4 Bouman Mental Health Care, Rotterdam, The Netherlands; Universidad de Granada, Spain

## Abstract

**Background:**

Although P300 amplitude reductions constitute a persistent finding in children of addicted parents, relatively little is known about the specificity of this finding. The major aim of this study was to investigate the association between parental rearing, adverse life events, stress-reactivity, substance use and psychopathology on the one hand, and P300 amplitude in response to both *target* and *novel* distracter stimuli on the other hand. Moreover, we assessed whether risk group status (i.e., having a parental history of Substance Use Disorders [SUD]) uniquely contributed to P300 amplitude variation above and beyond these other variables.

**Methods:**

Event-related potentials were recorded in high-risk adolescents with a parental history of SUD (HR;*n*=80) and normal-risk controls (NR;*n*=100) while performing a visual Novelty Oddball paradigm. Stress-evoked cortisol levels were assessed and parenting, life adversities, substance use and psychopathology were examined by using self-reports.

**Results:**

HR adolescents displayed smaller P300 amplitudes in response to novel- and to target stimuli than NR controls, while the latter only approached significance. Interestingly, the effect of having a parental history of SUD on *target-*P300 disappeared when all other variables were taken into account. Externalizing problem behavior was a powerful predictor of *target-*P300. In contrast, risk group status uniquely predicted *novelty-P300* amplitude reductions above and beyond all other factors.

**Conclusion:**

Overall, the present findings suggest that the P300 amplitude reduction to *novel* stimuli might be a more specific endophenotype for SUD than the *target-*P300 amplitude. This pattern of results underscores the importance of conducting multifactorial assessments when examining important cognitive processes in at-risk adolescents.

## Introduction

A wealth of data has shown that parental substance use disorders (SUDs) are associated with an array of long-lasting detrimental offspring outcomes, including early internalizing and externalizing behaviors [1,2], drug involvement when these offspring grow into adolescence [3], as well as an increased risk of developing (future) substance use-related problems [4]. Interestingly, both SUD patients and their offspring often display attentional difficulties [5,6]. The ability to selectively respond to relevant (environmental) cues while, at the same time, suppressing competing, spontaneous but inappropriate or irrelevant actions is crucial for adaptive functioning and goal-directed behavior [7]. Hence, impaired attentional control mechanisms (i.e., attentional orienting and selection) may be significant constituents of the multidimensional risk for developing a SUD [5]. Importantly, as attentional control deficits are related to an attenuation of the brain’s P300 event-related potential (ERP) amplitude, this neurobiological marker of attentional control has been proposed as a promising endophenotype for SUD [8].

The P300 refers to a positive deflection of the ERP arising about 300-800ms following the presentation of specific stimuli or events and the magnitude depends on the processing of the stimulus context and levels of attention and arousal [9]. Presented in the context of a three-stimulus novelty oddball paradigm in which infrequent, *non-target novel* stimuli are inserted into the sequence of infrequent *target* and frequent *standard* stimuli, the P300 contains two subcomponents: P3a and P3b [10,11,12]. Rare task-relevant *target* stimuli that require a specific response (i.e., button press) generate a parietal maximal P300 (P3b), whereas infrequent, non-repeating *novel* ‘distracter’ stimuli that are irrelevant for the task (i.e., no overt reaction is required) but are more salient than the targets usually elicit a P300 with a more central scalp topography (P3a). Theoretically, the *novelty-*P300 is supposed to reflect the automatic orienting response to new, salient stimuli. It marks the allocation of attentional resources to stimulus deviation and potentially significant events (i.e., an alerting process that originates when a distracting stimulus automatically demands focal attention) [13,14]. Conversely, the *target-*P300 is thought to reflect the neural mechanisms required to change the mental model of the environment (i.e., the updating of working memory) in order to respond appropriately, and the subsequent (effective) allocation of attentional resources to incoming task-relevant cognitive information [11].

With respect to SUD, P300 amplitudes in response to *targets* have been studied extensively. Target-P300 supposedly represents a vulnerability marker for SUD, as reduced P300 amplitudes have been repeatedly observed in high-risk (HR) offspring and other biological relatives of SUD patients (for reviews, see 15,16), though inter-study variation is also evident. In contrast, to date, *novelty-*P300 has barely been explored and only a few studies examined this novelty component in HR populations. There is evidence that both young and adult offspring of alcoholics manifest attenuated P300 amplitudes in response to infrequent novel non-target stimuli compared to normal-risk (NR) controls [17,18]. Although results have not been entirely consistent [19] and there is still some ground to cover (i.e., studies in a broad adolescent at-risk sample with a parental history of both alcohol and other substances of abuse are lacking), these findings suggest that the novelty-P300 may be a promising marker for vulnerability for SUD as well.

An important debate, however, concerns the origins of P300 aberrations in HR offspring. Twin and family studies have indicated that both target- and novelty P300 amplitudes are highly heritable [20], as heritability estimates range from 0.6 to 0.8 [21,22]. This may imply that the attenuated P300 amplitudes in HR adolescents can provide meaningful endophenotypic information concerning the genetic basis of these brain functions [23], and suggests that the observed deficits represent an inherited predisposition. However, since complex behaviors may also be influenced by past and current interactions within and across individuals and environmental contexts [24], there may be broader familial, environmental and behavioral factors that affect P300 outcomes in subsequent risk-group reports. 

It may be possible, for example, that the rearing environment provided by a SUD-diagnosed parent plays a role in developing attentional difficulties in their children. Substance abusers have impaired parenting skills which intensify the high-risk nature of the family environment [25], such as reduced parental monitoring [26] and less emotional warmth [27]. Moreover, offspring of addicted parents generally experience more traumatic, stressful and adverse life events than their peers without a parental history of SUD [28]. As the brain undergoes an intense period of maturation when children grow into adolescence [29,30], it seems plausible that early negative environmental experiences such as negative parenting and adverse life events can impact brain development and functioning. A few first empirical studies provide support for this view: In adolescents, higher levels of perceived emotional warmth prospectively predicted increased P300 amplitudes in response to positive feedback [31]. Moreover, a history of trauma predicted smaller P300 amplitudes to both target tones and distracting novel sounds in a sample of military cadets [32]. 

Another scarcely investigated variable that could influence the P300 amplitude is the stress response. Chronic life stressors may lead to blunted cortisol responses of the hypothalamic-pituitary-adrenal (HPA) axis system [33,34]. As stress is known to affect endocrine development and blunted cortisol levels may induce changes in brain function [35,36,37], this mechanism might also account for altered brain development, which, in turn, can lead to neuropsychological attentional deficits. In addiction research, it has been shown that stress is closely tied to the development and maintenance of SUDs [38]. Though results are not entirely consistent [39], numerous studies observed lower stress-evoked cortisol levels (i.e., HPA axis hypo-activation or hypo-arousal) in HR offspring as compared to controls [40,41,42]. Nevertheless, the exact role of early negative environmental experiences and the influence of the stress-evoked cortisol levels on P300 amplitudes has not been examined before.

Additional (behavioral) influences on P300 amplitudes may include adolescents’ habitual substance use tendencies as well as co-occuring psychopathology in HR offspring. More specifically, there is a considerable variance in frequency of substance use during adolescence and both target- and novelty P300 elicited by a visual three-stimulus oddball task appear sensitive to acute substance challenge, drug-use level, and drug type [43]. Both alcohol and nicotine use may result in diminished P300 amplitudes [43,44,45]. Chronic cannabis use, the most frequently used illicit drug [46], also influences both P300 amplitudes. However, overall *larger* amplitudes were observed in high-use individuals [43]. Since HR youth generally engage in greater amounts of substance use than the normal-risk controls and not every high-risk study excludes substance (ab)using participants, habitual substance use behavior may complicate the matter whether the observed deficits represent a vulnerability for, or a consequence of, substance use on the brain. In addition, the P300 variability from risk for SUD may be related to co-morbidity for a broader spectrum of externalizing problems and disinhibited psychopathologies [47,48,49]. Hence, reduced P300 amplitudes found within populations of individuals at high-risk for SUD may not be specific to SUD. Rather, P300 amplitude may only be significantly reduced among HR adolescents when the sample includes individuals with comorbid externalizing symptoms or disorders. 

Taken together, there may be multifactorial determinants of target- and novelty-evoked P300 amplitudes in children of addicted parents, including influences of parental rearing, adverse life events, stress reactivity, substance use, and psychopathology. However, as far as we know, there are no studies to date that have explored simultaneously the relative contributions of these variables to explain the magnitude of target and novelty-evoked P300 amplitudes. Moreover, the existing literature has not evaluated the unique contribution of having a parental history of SUD on P300 amplitudes. Therefore, in the present study, we examined P300 amplitudes in response to task-relevant target and irrelevant novel stimuli during a visual three-stimulus oddball task in a sample of HR and NR adolescents. The main aim was to evaluate the potentially predictive value of the above-named multifactorial determinants on P300 amplitude responses, and to assess whether risk group status uniquely predicted target and/or novelty P300 amplitude variation above and beyond the other factors.

## Methods

### Participants

A total sample of 83 high-risk (HR) and 110 normal-risk (NR) adolescents between the ages of 12 and 20 years old was recruited for the present study (Youth in the Netherlands Study [JOiN]; [50]. For the current analyses, data of 12 participants were not included due to EEG measurement errors (i.e., they had less than 50% artifact-free epochs of the total number of ERP segments or less than 15 artifact-free *target* ERP epochs; *n*=11; 3 HR adolescents and 9 NR adolescents), or because none of the (background) questionnaire data were available (1 NR adolescent), resulting in a final sample of 80 HR and 100 NR adolescents.

#### High Risk group

The HR adolescents (*n* = 80; 39 males; mean age = 15.5, *SD* = 2.4) consisted of adolescents who were recruited via their parents treated at the outpatient clinics of Bouman Mental Health Care (Rotterdam, the Netherlands). All parents had been diagnosed with and treated for a DSM-IV-TR diagnosis of a substance use disorder (SUD) other than nicotine. Diagnosis of SUD in participants’ parent was based on information from the medical records. Treatment staff informed eligible patients, gave them an information brochure and both patients and their children were asked to participate. After permission of both parents and their children, participants were screened by telephone and after confirmation of eligibility, an appointment for the test session was made. Six HR adolescents were recruited through an outpatient Youth clinic of Bouman GGZ, being in treatment themselves and their parents were known to have a diagnosis of SUD (these adolescents had already developed cannabis-related problems and were treated for a DSM-IV-TR diagnosis of cannabis abuse and/or dependence). Moreover, 6 other HR participants had parents who were diagnosed with a SUD but were not currently in treatment. These participants were recruited by word of mouth referral, and SUD diagnosis in the parents was ascertained via a structured interview (CIDI; Robins et al., 1989), performed by a trained interviewer of the research staff prior to participation of the offspring in the study.

Of the included HR adolescents, 44 (55.0%) had a father with a SUD diagnosis, 35 (43.8%) had a SUD diagnosed mother, and in one case both parents had a lifetime DSM-IV-TR diagnosis of SUD (1.3%). The pattern of parental SUD diagnoses was heterogeneous. The most prevalent SUD diagnosis was alcohol abuse and/or dependence (*n*=54; 67.5%), followed by abusing/dependency of cannabis (*n*=4; 5.0%), cocaine (*n*=3; 3.8%) and sedatives (*n*=2; 2.5%). Sixteen parents had more than one SUD diagnosis and were using a combination of two or more substances; 20.0%). Data on drug use of one parent were missing.

#### Normal risk group

Adolescents in the community-based NR group (*n*=100; 53 males; mean age = 15.0, *SD* = 2.0) were part of a larger sample that participated in a general population study (*n* = 2567) of youth aged 6 to 20 years old [51], see Huizink et al. [50] for further details. The NR adolescents included in the present study were randomly ascertained from the larger sample with the only condition that they were in the targeted age range (12-20 years). A psychiatric disorder in the parent as well as in the adolescent did not disqualify the adolescent for participation in the study in order to maximize the representativeness of the sample.

All adolescents included in the study were fluent in Dutch, were physically healthy, and had no history of head injury, mental retardation, or neurological disorders. Written informed consent was obtained from all parents and adolescents before their participation. The research protocol was approved by the Medical Ethical Committee of the Erasmus Medical Center Rotterdam, The Netherlands. The study was conducted in accordance with the declaration of Helsinki.

### Measures

#### Target- and Novelty-P300 as an index of attentional control

**Figure 1:**
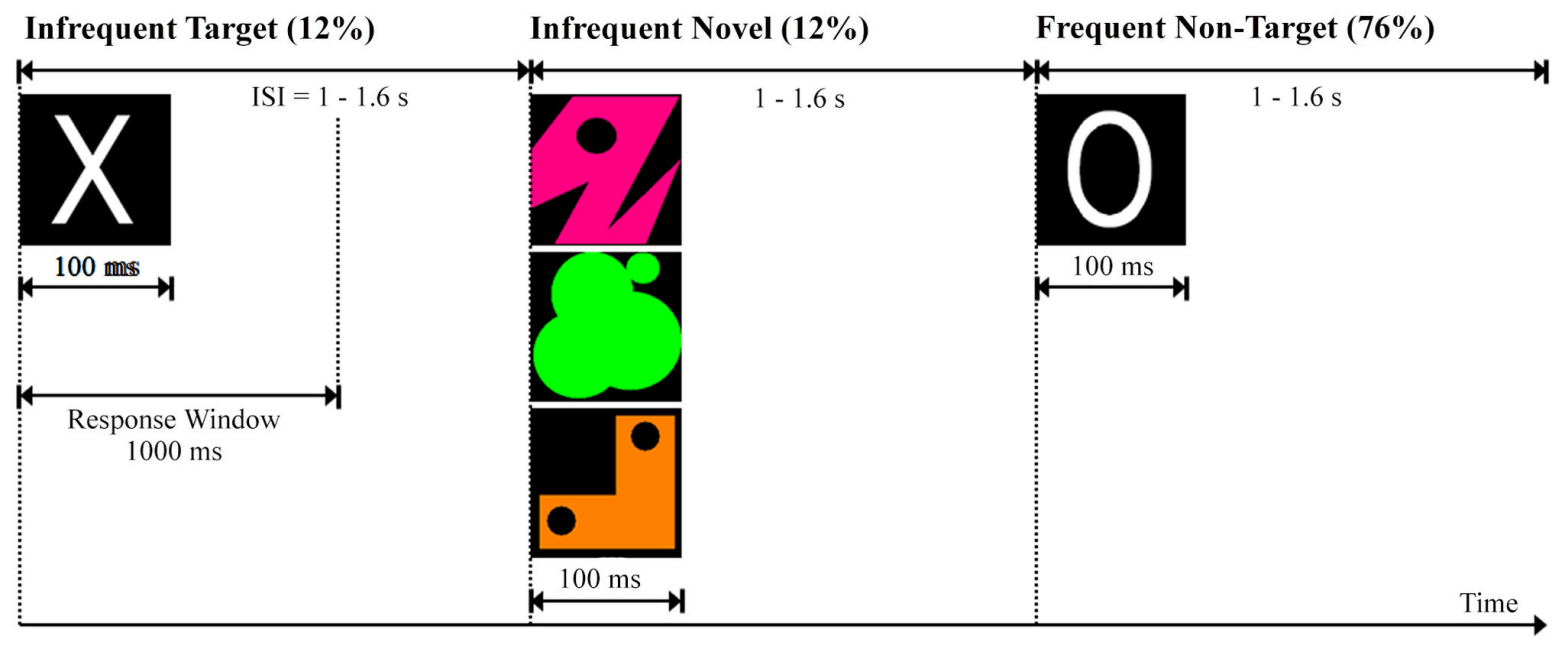
Illustration of the visual event-related oddball design (derived from Jones et al., 2006).

Visual P300 amplitudes of the ERP were elicited using a modified version of the Visual Novelty Oddball paradigm as has been previously described by Van der Stelt et al. [18,52]. Adolescents were exposed to three types of visual stimuli: 1] frequently occurring non-target stimuli (a white letter O), 2] rare target stimuli (a white letter X), and 3] rare novel stimuli (unique, non-repeating colorful abstract patterns, e.g., blue triangles, yellow circles, blue pentagons, etc.), see Figure 1. Novel stimuli were purposefully designed so that each occurrence was a unique perceptual event (in order to elicit a robust non-target P300 response). All stimuli were presented in the middle of a black computer screen positioned at circa 110 cm from the adolescent, for the duration of 100ms with a variable interstimulus interval to minimize habituation. The ISI range was 1-1.6s. Stimuli were presented pseudorandomly, with the constraints that the first 5 stimuli within each block were always standard non-targets and that neither targets nor novels could be repeated in succession. After one block of 50 practice trials, including target (12%) and non-target (88%) stimuli, subjects received four blocks of experimental trials, each containing 100 stimuli, including non-target (76%), target (12%), and infrequent novel (12%) stimuli. Altogether, in the experimental session 400 stimuli were used, including 304 *standard non-target* stimuli, 48 *target* stimuli and 48 *novel* stimuli. Participants were instructed to respond as quickly as possible, but not at the cost of accuracy, whenever a target stimulus was detected with a button press response with the index finger of their dominant hand and to refrain from responding when the standard or novel stimuli were presented. They were not informed about the presentation of the novel stimuli in the experimental session. By use of this visual P300 paradigm, both the P300 obtained actively by the *target* stimuli (i.e., target-P300) and the P300 response elicited passively by the irrelevant but attention-capturing *novel* stimuli (i.e., novelty-P300) could be investigated.

Although we had no specific research question or hypothesis about the performance measures (RT and errors), we additionally analyzed the behavioral measures for possible guidance in the interpretation of the P300 results.

#### Parental rearing behavior

Perceived parental rearing behaviour was assessed with the EMBU-C (Egna Minnen Beträffende Uppfostran; a Swedish acronym for My Memories of Upbringing; [53]), a dimensional instrument that measures the child’s perception of his or her upbringing. The version we used is to a large extent in accordance with the Dutch version of the EMBU developed by Markus et al. [54], including items on rejection, emotional warmth, overprotection and favoring subject. In the present study, the questionnaire was changed in two ways. First, all items referring to siblings were omitted because not all children had siblings (removing one item in the rejection subscale). Second, we did not use the favoring subject scale for our analyses because of its reported low internal consistency [55]. As a result, the EMBU-C version that was used in the present study contained 47 items in total that were answered on a four-point likert scale (*1 = no, never; 2 = yes, but seldom; 3 = yes, often; 4 = yes, most of the time*). For the current analyses, the judgments of the rearing behaviors of fathers and mothers were averaged into single measures of parental rejection (RE), emotional warmth (EW) and overprotection (OV). 

#### Adverse life events

Adverse Life Events (ALE) were selected from an extensive Life Events Questionnaire (LEQ; [56]) which includes both severely and mildly adverse events as well as positive events, and from the post-traumatic stress disorder section of the NIMH Diagnostic Interview Schedule Composite (DISC). Eighteen severely adverse events were chosen from these sources, modelled as closely as possible after Lovallo et al. [57]. Both the DISC interview and the LEQ were completed by the adolescent and his/her parent. An event was considered an ALE if either the parent or adolescent confirmed that the event was experienced by the adolescent. For the LEQ, events were only considered an ALE if the informant coded the event as ‘unpleasant’ (for the adolescent). For the present study, ALEs were summed.

#### Stress-evoked cortisol levels

In order to measure stress reactivity, stress-evoked cortisol levels were examined. Stress procedure sessions commenced with an explanation of the procedure by the experiment leader. After the completion of two questionnaires and a ten minute pre-task rest period, the social stress tasks began, which were characterized by uncontrollability and social-evaluative threat, thus designed to elicit a stress reaction [58]. These tasks entailed a mental arithmetic task (4 min), a public speaking task (8 min mental preparation, 6 min speech) and a computer mathematics task (5 min; see 59 for full details on the procedure). The session ended with a five minute recovery period and a relaxing nature documentary (25 min). After each period/task, at the middle of the movie and at the end of it, the participant was asked to provide saliva samples (6 samples). These samples reflect activity in the hypothalamus approximately 20 minutes earlier due to the delay in observable cortisol response [60]. Saliva samples were kept in a freezer at -20 degrees Celsius [61] and were collectively sent to the laboratory for analysis. A time-resolved fluorescence immunoassay was implemented to determine the cortisol concentration (details available upon request). Outliers greater than 3 SD above the mean were removed from the analysis because of possible contamination (e.g. blood, medicine).

To assess HPA-axis reactivity (i.e., cortisol levels in response to stress), we calculated the area under the curve with respect to increase (AUCi; [62]. For this, we excluded the first cortisol pre-task value from the analyses as it was generally higher, thus most likely reflecting anticipatory stress to a greater degree than the second measurement. Five cortisol samples were thus used in the calculation of the AUCi. Preliminary exploratory analyses showed a curvilinear relation between cortisol and P300 amplitudes, therefore we standardized the AUCi variable and used this to create three groups of individuals showing normal stress (standardized AUCi between -1 and 1 SD) and individuals showing dysregulated stress, stratified into hypo-arousal (standardized AUCi less than -1 SD) and hyper-arousal (standardized AUCi greater than 1 SD). 

#### Habitual substance use behavior

A self-report Substance Use Questionnaire (SUQ; [63]) was used to assess adolescents’ early-onset exposure to and experimentation with alcohol, tobacco, and cannabis use. Frequency of substance use was examined for the total sample by calculating the number of drinks/use per week. 

#### Psychopathology

The *Youth Self-Report questionnaire* (YSR [64]; was used to assess self-reported behavioral problems or psychopathology. Six subscales were calculated on the basis of *Diagnostic and statistical manual of mental disorders* (DSM-IV-TR; [65]) diagnoses. Scores on three of these subscales (Affective, Anxiety and Somatic disorders) were summed in order to obtain a general score of number of Internalizing symptoms. A general score for number of Externalizing symptoms was similarly achieved (using subscales Attention deficit hyperactive, Conduct and Oppositional defiant disorders). The YSR has a good validity and test-retest reliability [66]. 

### Procedure

Eligible participants were invited to the Erasmus Behavioral Lab (Erasmus University Rotterdam). At arrival, participants gave their written informed consent and completed several self-report questionnaires with respect to behavioral traits as well as their usual substance use behavior. All participants then took part in an EEG session, lasting approximately 75 minutes in total. Participants were seated on a comfortable chair in a light and sound-attenuated room. After the EEG electrodes were attached, the Visual Novelty Oddball paradigm was administered (~ 15 minutes). Subsequently, participants completed two other tasks (not reported in this paper). Hereafter, HR participants consecutively took part in another laboratory protocol (i.e., stress-reactivity session) that was part of the larger JOiN project [50], where saliva samples were collected in order to evaluate stress-evoked cortisol levels. For NR adolescents, this stress-reactivity session had been conducted during a prior assessment. Thus note that data regarding parental rearing behaviors and (environmental) stress, including saliva samples were obtained in the NR group during a visit prior to the EEG session, whereas these data in the HR group were collected after the EEG session. All adolescents received a gift certificate for their participation. 

### Electroencephalogram (EEG) acquisition and analysis

The EEG was recorded with BioSemi Active-Two using 34 scalp sites (10-10 system, and two additional electrodes at FCz and CPz) with Ag/AgCl active electrodes mounted in an elastic cap. Six additional electrodes were attached: two to the left and right mastoids as reference electrodes, two were placed next to each eye for the horizontal electrooculogram (HEOG) to record ocular movement and to be able to correct for ocular artifact, and two electrodes were placed above and below the left eye for vertical electrooculogram (VEOG). Online signals were recorded with a low-pass filter of 134Hz. All signals were digitized with a sample rate of 512Hz and 24 bit A/D conversion. 

Data were off-line referenced to mathematically linked mastoids. A conventional wide band filter of 0.1 to 30 Hz (phase shift-free Butterworth filters; 24dB/octave slope) was used. Data were segmented in stimulus-locked epochs of 900ms (100ms pre-stimulus until 800ms post-stimulus). After ocular correction [67], epochs including out of range voltages (±100µV) were rejected as artifacts and were excluded from further processing. The mean 100ms pre-response period served as baseline. After baseline correction, epochs locked to target, novels and standard stimuli were averaged separately for artifact-free trials at each scalp site, producing one average waveform per stimulus condition per participant. The mean number of included *target* trials was 39.61 (*SD* = 7.42; 82.5% of all epochs), and mean number of *novel* trials was 42.02 (*SD* = 5.52; 87.5% of all epochs). The mean number of available epochs did not differ between groups (both *p*’s >.99).

P300 amplitudes in response to target and novel stimuli were identified as the mean value within a 350 to 700ms window following stimulus onset, derived from inspecting grand average and individual subject data. This area measure is less sensitive to noise than simply assessing the maximum peaks of a component [68]. For the purpose of statistical analyses, we focused on the target-P300 amplitudes on the parietal midline electrode Pz, as P300 amplitude is generally largest on this electrode and in order to compare our results with previous studies (most studies report only Pz). The novelty-P300 is less examined, and there is more variation in distribution, therefore the novelty-P300 was assessed by focusing on the three midline electrodes Pz, CPz and Cz.

### Statistical analyses

To calculate pre-existing group differences regarding demographic characteristics, scores on the subjective self-report ratings (i.e., psychopathology, parental rearing, life events and frequency of substance use) and stress-evoked cortisol levels, independent samples t-tests and chi-square tests were used. Behavioral performance was evaluated by using ANOVA’s with Group (HR vs. NR) and Gender (male vs. female) as between-subject factors and consecutively the RT’s on correct identified targets, the percentage of correct responses and error rates as dependent variables.

All other analyses were conducted for target- and novelty P300 amplitudes separately. First, to evaluate the influence of risk group status on P300 amplitudes and to explore possible gender differences, a Group x Gender ANOVA was performed for the P300 in response to targets, and a 2 (Group) x 2 (Gender) x 3 (Electrode site: Pz, CPz and Cz) repeated measures ANOVA for the P300 in response to novel stimuli. Greenhouse-Geisser corrections were adopted where appropriate (uncorrected df’s are reported) and all significant ANOVA effects were further analyzed using Bonferroni-corrected post-hoc t-tests. Second, bivariate correlation analyses using Pearson’s correlation coefficient were computed across groups to examine associations between the P300 amplitudes and all independent variables. Third, through use of hierarchical multiple regression analyses we assessed the determinants of P300 amplitude reductions in response to target as well as novel stimuli, and we examined whether risk group status (i.e., having a parental history of SUD) explained a unique proportion of the variance in P300 above and beyond the other variables. For this purpose, the regression analyses were performed in three steps, resulting in 3 models. First, the demographical information of the participants (covariates; i.e., age and gender) was entered in step 1. Second, all variables hypothesized as significantly contributing to P300 amplitudes were simultaneously entered in Step 2, i.e., perceived parental rearing behaviors (emotional warmth, rejection and overprotection), adverse life events, stress-evoked cortisol levels, frequency of substance use, and psychopathology (internalizing and externalizing problem behavior). As stress-evoked cortisol represents a categorical variable with three levels, two dummy variables were created: (1) normal stress vs. underarousal; and (2) normal stress vs. hyperarousal. Finally, in step 3, risk group status (HR vs. NR) was entered into the equation to examine its unique contribution to the model while controlling for the previously entered variables. All predictors were checked for multicollinearity by means of tolerance statistics. Values of the Variance Inflation Factor (VIF) were all well below 10 (i.e., all less than 2.9) and tolerance statistics all well above 0.2 (i.e., all > .35); indicating that there is no collinearity within our data (e.g.,[69]). For all analyses, a .05 level of significance was employed. 

## Results

### Sample characteristics

Table 1 shows the descriptive information with respect to demographics, parental rearing, adverse life events, stress-reactivity, substance use behavior and psychopathology of the HR and NR adolescents. Adolescents in both groups were comparable in age (*p*=.13) and gender (*p*=.57). However, HR adolescents experienced less emotional warmth than NR controls (*p*<.01). There was also evidence of an increased number of adverse life events and reduced levels of stress-reactivity (i.e., hypo-arousal) in the HR group (all *p*’s<.001). Assessment of behavioral traits (psychopathology and frequency of substance use) revealed that HR adolescents scored higher on both Externalizing and Internalizing symptoms (both *p*’*s*<.001), as well as on Frequency of nicotine and cannabis use (both *p*’s<.05) than NR controls. Note, however, that while HR adolescents smoked more cannabis, the frequency was relatively low (i.e., less than once a week).

**Table 1:** Characteristics of the HR and NR adolescents.

		**HR (*n* = 80)**				**NR (*n* = 100)**					
	***N***	**Mean or frequency (%)**	**SD**		***n***	**Mean or frequency (%)**	**SD**		***t***	**X^2^**	***p***
Demographics											
Age (in years)	80	15.51	2.43		100	15.00	2.01		-1.52		.13
Gender (% males)	80	53%			100	48.8%				0.32	.57
Parental Rearing Behaviors											
Rejection	72	1.47	0.69		99	1.40	0.29		-0.92		.36
Emotional Warmth	73	3.00	0.82		99	3.29	0.50		2.83		.005
Overprotection	72	1.88	0.35		99	1.83	0.32		-1.00		.32
Adverse Life Events (sum)	80	3.83	2.39		100	1.54	1.28		-7.72		<.001
Stress-reactivity (AUCi 6-10)	71	201.30	146.28		80	306.47	113.13		4.97		<.001
Hypo-arousal	24	33.8%			4	5.0%				20.72	<.001
Normal arousal	38	53.5%			60	75.0%					
Hyper-arousal	9	12.7%			16	20.0%					
Substance use (per week)											
Number of drinks	78	4.23	5.66		92	3.22	5.68		-1.16		.25
Number of cigarettes	78	21.29	42.43		95	7.42	25.07		-2.55		.012
Cannabis use	78	0.11	0.25		94	0.01	0.02		-3.55		.001
Psychopathology											
Externalizing	76	1.71	0.85		94	0.82	0.65		-7.45		<.001
Internalizing	71	1.10	0.83		94	0.55	0.61		-4.80		<.001

### Behavioral performance

Behavioral data of two participants (1 HR and 1 NR participant) were not available. With respect to accuracy (correct response rate to targets), both HR adolescents and NR controls performed well (mean accuracy HR group = 98.9% (SE = 0.32); mean accuracy NR group = 99.4% (SE=0.28) and there was neither a main effect of group (F(1,174)=1.35, p=.25), nor gender (F(1,174)=0.22, p=.64). False alarm rates were negligible (all < 2%; HR = .05 % vs. NR = .10% for infrequent non-targets and HR = .97 % vs. NR = 1.37 % for infrequent novel stimuli) and did not differ among Groups (both p’s > .08) or Gender (both *p*’s>.39). No significant Group x Gender interaction-effects were observed (both p’s > .20). Analyses of response time (RT) data revealed a main effect of Group (*F*(1,174)=4.19,*p*=.04), indicating that the HR group responded faster (mean RT = 375ms, SE = 5.2ms) than the NR controls (mean RT=389, SE=4.6ms). We also observed a main effect of Gender (*F*(1,174)=5.02,p=.03). Overall, male adolescents were faster in detecting the targets than females (374ms vs. 390ms, respectively). No interaction-effect could be observed (*p*=.31). 

### Target-P300 amplitudes

#### Risk Group status effects

**Figure 2:**
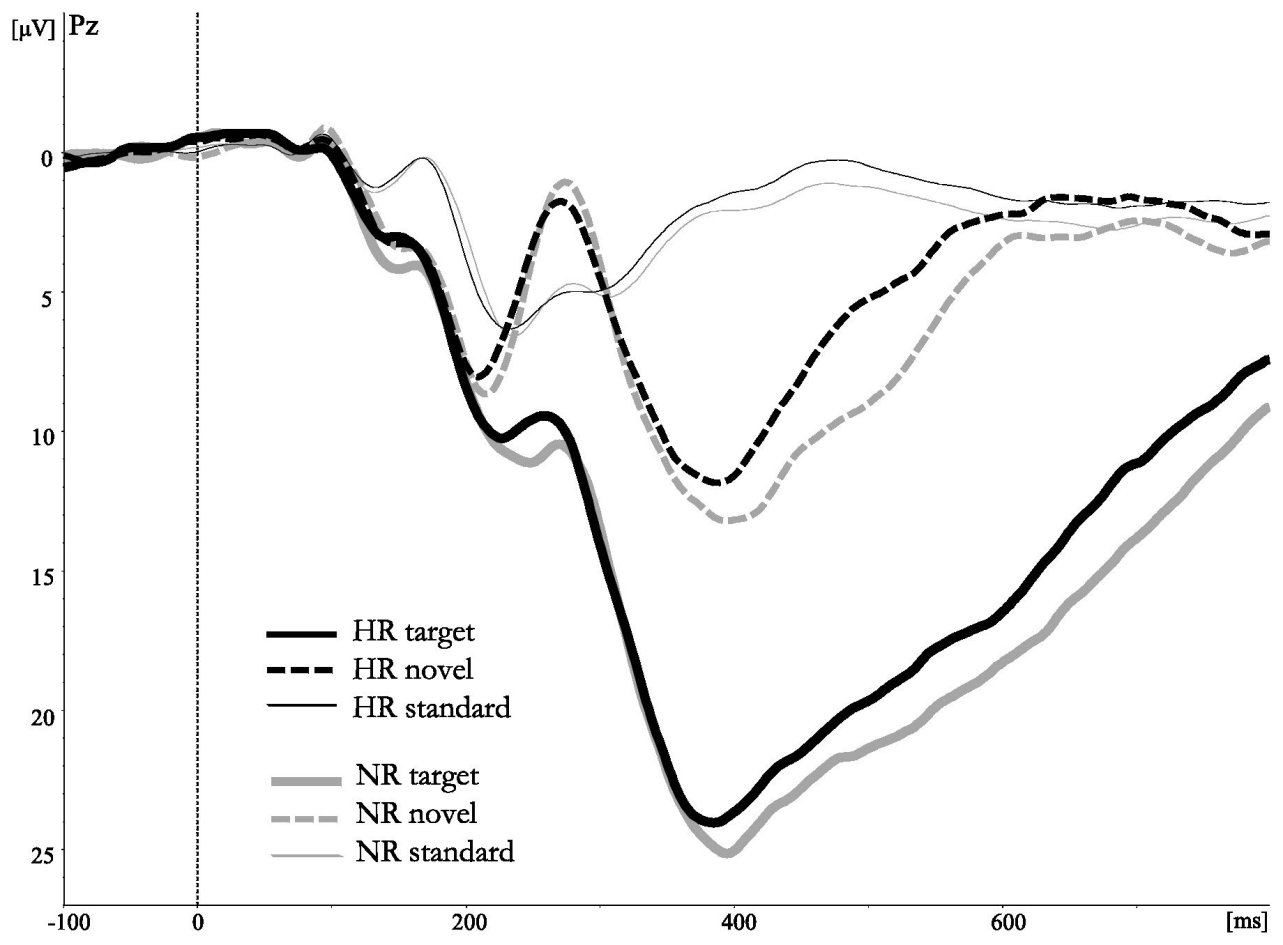
Stimulus-locked grand average waveforms (filtered 0.1-30 Hz) from electrode site Pz evoked by frequent non-target stimuli, infrequent target- and infrequent novel stimuli.

Figure 2 shows the grand average ERP waveforms to non-target, target and novel stimuli at the midline electrode site Pz for the group HR adolescents with a parental history of SUD and NR controls. Table 2 presents means and standard deviations of P300 amplitudes in response to both targets and novelty stimuli. HR adolescents tended to display lower target-P300 amplitudes as compared to NR controls (18.6μV vs. 20.7μV, respectively), however, repeated measures ANOVA revealed that the main effect of Group only approached significance (*F*(1,176)=3.18, *p*=.076). Neither the main effect of Gender (mean males: 18.7μV, mean females: 20.5μV; *F*(1,176)=2.45, *p*=.12), nor the Group x Gender interaction-effect reached significance (*F*(1,176)=0.51, *p*=.48).

**Table 2:** Means and Standard deviations of target- and novelty-P300 amplitudes in HR and NR adolescents.

			**HR (*n*=80)**						**NR (*n*=100)**				
			**Male (*n*=39)**			**Female (*n*=41)**			**Male (*n*=53)**			**Female (*n*=47)**	
			*Mean*	*SD*		*Mean*	*SD*		*Mean*	*SD*		*Mean*	*SD*
**Target-P300**	Pz		18.1	8.8		19.1	7.5		19.3	6.9		22.0	8.1
**Novelty-P300**	Cz		2.4	5.3		2.1	5.8		3.4	6.7		4.0	5.4
	CPz		4.3	4.9		4.1	6.0		5.9	6.4		6.2	4.7
	Pz		6.1	4.4		5.0	5.5		7.6	6.5		7.6	4.1

#### Correlational analysis

Table 3 presents the correlations between the P300 amplitudes and all independent variables. P300 in response to target stimuli was inversely related to age (*r*=-.23, *p*=.002), indicating that the target-P300 amplitude diminished with increasing age. Perceived emotional warmth was positively associated with the target-P300 (*r*=.18, *p*=.02), whereas both frequency of alcohol use (*r*=-.18) and smoking (*r*=-.16) were negatively related to target-P300 (both *p*’s<.05). Similarly, target-P300 was inversely correlated to both hypo-arousal and externalizing problem behavior (*r*=-17, *p*<.05 and *r*=-.21, *p*<.01, respectively).

**Table 3:** Correlations between dependent and independent variables across groups.

	**Target-P300**	**Novelty-P300**
Group	-.13	-.19*
Age	-.23**	-.02
Gender	.12	-.05
EW	.18*	.09
OP	-.01	.04
RE	-.06	.02
Freq Alcohol	-.18*	-.04
Freq Nicotine	-.16*	.01
Freq Cannabis	-.13	-.01
ALE	.01	-.04
Stress AUC	.11	-.02
*normal stress (0*)* vs. hypo-arousal (1*)	-.17*	-.09
*normal stress (0*)* vs. hyper-arousal (1*)	-.01	-.12
Internalizing	.01	-.08
Externalizing	-.21**	-.23**

Note: Group = risk group status: 0 = NR, 1 = HR; Gender: 0 = male, 1 = female; EW = emotional warmth; RE = rejection; OP = overprotection; Freq = frequency of substance use (number of drinks/use) per week; ALE = adverse life events; Stress AUC = stress-evoked cortisol levels (area under the curve tube 6-10): 0 = hypo-arousal, 1 = normal arousal, 2 = hyper-arousal. * *p* < .05; **p < .01.

#### Predictors of task-relevant target-P300

Results of the hierarchical multiple regression analysis on target-P300 amplitudes are presented in Table 4. The overall R^2^ in the final model, when all variables hypothesized as significantly contributing to the magnitude of target-P300 were considered, was significant, *F*(_14, 111_) = 2.35, *p*=.007), accounting for 23% of the variance. Neither parental rearing behaviors, nor frequency of substance use significantly contributed to the variance in target-P300. Note that while cortisol levels only approached significance in the final model (*p*=.06), hypo-arousal (i.e., HPA-axis hypo-activation) significantly predicted target-P300 amplitude reductions in model 2. In the final model, adolescents’ age (β=-.25, SE=.40, *p*=.02) and the number of adverse life events (β=.21, SE=.46, *p*=.05) were significant predictors. Moreover, externalizing problem behavior was a powerful contributor to the prediction of target-P300 amplitude (β=-.31, SE=1.16; *p*=.01), indicating that adolescents who reported higher levels of externalizing symptoms displayed smaller target-P300 amplitudes. Importantly, while controlling for the previously entered variables, risk group status (i.e., having a parental history of SUD) did not explain a unique proportion of the variance above and beyond these other factors, i.e., group was not a significant predictor of target-P300 amplitude (β=.02, SE=1.99, *p*=.87).

**Table 4:** Hierarchical Multiple Regression Analysis on target-P300 amplitudes (*n* = 126).

**Predictor variable**	**Model 1**	**Model 2**	**Model 3**
*Demographics*			
Age	-.25**	-.25*	-.25*
Gender	.15	.16	.16
*Parental rearing*			
RE		.11	.11
EW		.15	.15
OP		-.04	-.04
*Adverse life events*		.21*	.21*
*Stress reactivity*			
Hypo-arousal		-.19*	-.20
Hyper-arousal		-.06	-.06
*Substance use behavior*			
FreqAlc		.01	.01
FreqNic		-.02	-.02
FreqCan		-.01	-.01
*Psychopathology*			
Internalizing		.11	.11
Externalizing		-.31**	-.31**
*Risk group status*			
Group			.02
**R^2^**	.08**	.23**	.23**
**ΔR^2^**		.15*	.00

*F*(_14, 111_) for the entire model = 2.35, *p* = .007.

Note: Standardized regression coefficients (or *β* weights) are presented; *p <.05. ** *p* <.01.

### Novelty-P300 amplitudes

#### Risk Group status effects

Repeated measures ANOVA revealed a significant main effect of Group (*F*(1,176)=4.82, *p*=.03), showing that HR adolescents displayed lower novelty-P300 amplitudes as compared to NR controls (4.0μV vs. 5.8μV, respectively). Furthermore, a main effect of electrode site (*F*(2,352)=160.47, *p*<.001) emerged, with increasing amplitudes from central to parietal sites (Cz = 3.0μV, CPz = 5.2μV and Pz = 6.6μV, respectively). Neither the main effect of Gender (*F*(1,176)=0.02, *p*=.90), nor the interaction-effects including the factors Group or Gender reached statistical significance (all *p*’s>.19). 

#### Correlational analysis

P300 in response to novel distracter stimuli appeared to be only significantly negatively correlated to risk group status (*r*=-.19, *p*=.01) and externalizing problem behavior (*r*=-.23, *p*=.002). Neither frequency of substance use, nor environmental factors or stress reactivity were associated with the magnitude of novelty-P300.

#### Predictors of task-irrelevant novelty-P300

Table 5 presents the results of the hierarchical multiple regression analysis on novelty P300 amplitudes at Pz (where the amplitude was maximal). Results indicated that the model- and change statistics of the first two models did not reach statistical significance (all *p*’s>.20). In model 3, risk group status was entered, resulting in a 0.05 increment with demographics, parenting, (environmental) stress, substance use behaviors and psychopathology held constant (*F* ^change^(1,111)= 7.00, *p*=.009). Together, this final model significantly explained a total of 18.7% of the variance in novelty-P300 amplitudes (*F*(_14, 111_) = 1.82, *p*=.04). There were only two significant predictors. First, externalizing problem behavior was significant (β=-.24, SE=.76, *p*=.05), indicating that adolescents who reported higher levels of externalizing symptoms displayed smaller novelty-P300 amplitudes. Most importantly, when taking all other variables into account, risk group status appeared to be a strong contributor to the prediction of novelty-P300 (β=-.32, SE=1.29, *p*=.009). These results showed that having a parental history of SUD is associated with smaller P300 amplitudes in response to novel stimuli. Hence, risk group status explained a unique proportion of the variance in novelty-P300 above and beyond all other variables. 

**Table 5:** Hierarchical Multiple Regression Analysis on novelty-P300 amplitudes (*n* = 126).

**Predictor variable**	**Model 1**	**Model 2**	**Model 3**
*Demographics*			
Age	-.14	-.11	-.12
Gender	-.08	-.08	-.10
*Parental rearing*			
RE		.13	.12
EW		.02	.00
OP		-.04	-.05
*Adverse life events*		.06	.17
*Stress reactivity*			
Hypo-arousal		-.15	-.03
Hyper-arousal		-.14	-.12
*Substance use behavior*			
FreqAlc		-.06	-.08
FreqNic		-.07	-.06
FreqCan		.02	.09
*Psychopathology*			
Internalizing		.12	.13
Externalizing		-.29*	-.24*
*Risk group status*			
Group			-.32**
**R^2^**	.03	.14	.19*
**ΔR^2^**		.11	.05**

*F*(_14, 111_) for the entire model = 1.82, *p* = .044.

Note: Standardized regression coefficients (or *β* weights) are presented; *p <.05. ** *p* <.01.

## Discussion

Our study examined simultaneously the hypothesized relative contributions of parental rearing behavior, adverse life events, stress-reactivity, habitual substance use, and (externalizing) psychopathology on P300 amplitude variation in a sample of high-risk (HR) adolescents who are thought to be at increased risk for the development of (future) substance use-related problems because of a parental history of SUD and normal-risk controls (NR) without such history. Moreover, to our knowledge, the current study is the first that examined the unique contribution of having a parental history of SUD on P300 amplitudes after taking into account the broader familial, environmental, and behavioral (risk) factors. Our major finding is that the effect of having a parental history of SUD on *target-*P300 disappeared when the variables hypothesized as important contributors were taken into account. In contrast, risk group status uniquely predicted *novelty-*P300 amplitude reductions above and beyond all other factors. 

The characteristics of the study group emphasize the importance of considering familial, environmental, physiological and behavioral background variables in at-risk adolescents. HR offspring reported experiencing less parental emotional warmth (i.e., a style of parenting that is characterized by showing unconditional love, given special attention, praising approved behavior, and being supportive and affectionately demonstrative) and an increased number of adverse life events than NR controls, which is in line with previous research [27,28]. In addition, we observed lower stress-evoked cortisol levels in the HR group. This arousal pattern has been proposed to be an alternative indicator of an inborn vulnerability (or endophenotype) to the development of SUDs [38]. Assessment of behavioral traits (psychopathology and frequency of substance use) further revealed that HR adolescents scored higher on both externalizing and internalizing symptoms as well as on frequency of nicotine and cannabis use than NR controls. Together, these findings confirm the high-risk status of our HR sample and again demonstrate that parental SUDs are related to an array of unfavorable offspring outcomes. 

With respect to the target-P300, multiple hierarchical regression analysis revealed that risk group status (i.e., having a parental history of SUD) was no longer related to target-P300 amplitudes when all variables hypothesized as significantly contributing to the P300 magnitude were taken into account. In contrast, externalizing problem behavior appeared to be a powerful contributor to the prediction of target-P300, indicating reduced target-P300 amplitudes among adolescents with increased levels of externalizing problem behavior. This finding corroborates previous research demonstrating P300 amplitude reductions among teenagers with a conduct disorder or other externalizing symptoms [70,71,72,73], whereas having a family history of SUD, even in individuals from more densely affected families, had no significant effects [71]. Externalizing problems and (risk for) substance use frequently co-occur, and there is well-replicated evidence for the existence of a coherent genetic externalizing liability, with a shared variance across broad externalizing symptoms that also includes SUDs [74,75,76,77]. The present finding thus lends support to the hypothesis that SUDs are part of a broader externalizing spectrum [78], and that reduced target-P300 amplitudes merely represent a general vulnerability for this broader externalizing spectrum, rather than a specific risk factor for SUD [72,78,79]. The results of the present study suggest that a family history of SUD is neither a required nor sufficient cause of P300 amplitude reductions in adolescents [71]. Rather, P300 reductions previously attributed to a positive family history may be the result of undiagnosed externalizing problems, such as conduct attention-deficit/hyperactivity (ADHD), oppositional defiant (ODD), and conduct (CD) disorders [72]. This may also elucidate the inconsistency between the absence of a family history effect in some studies [71] and significant risk group status effects reported in other studies (for reviews, see 15,16. Arguably, studies that report an association between family history and reduced target-P300 amplitudes may be confounded by the pronounced effects of other externalizing problems. Hence, we encourage future high-risk studies to assess comorbid childhood externalizing symptoms, in order to avoid the risk of failing to recognize an important mediating variable.

Regression analysis further denoted the importance of environmental influences on target-P300 amplitudes, as adverse life events (ALE) significantly, albeit moderately, predicted the magnitude of P300. More specifically, the experience of more adverse life events was associated with *larger* target-P300 amplitudes. This was a rather unexpected finding given that stress and ALE generally have been adversely associated with cognitive functioning [80,81]. Given the fact that bivariate analyses revealed no zero-order correlation between ALE and the target-P300 amplitude, it is likely that our finding is due to an artifact of the multiple regression analysis. Specifically, there may be evidence of a suppressor effect [82,83], indicating that although ALE and target-P300 were uncorrelated, the prediction in target-P300 increases when ALE is added to the equation simply because this suppressor variable is correlated with another predictor (or set of predictors) that are correlated with target-P300. The present finding should thus be interpreted with caution and this issue awaits further investigation. 

Interestingly, though only marginally significant in the final model of the multiple hierarchical regression analysis, lower stress-evoked cortisol levels (*hypo-active* HPA-axis in response to stress) significantly predicted reduced target-P300 amplitudes in model 2 (i.e., in the step before risk group status was entered into the model). Research has examined reduced P300 amplitudes and blunted cortisol levels (i.e., dysregulated stress reactivity) as hypothesized endophenotypes separately in SUD patients as well as in their children [40]. However, attempts to identify *multivariate* endophenotypes for SUD using these two measures together have not been revealed yet. Nevertheless, our study suggests that the two measures are related, and that hypo-reactivity (hypo-arousal) in particular is able to predict reduced target-P300 amplitudes. Hence, this would be an importing starting point for future studies.

With respect to P300 amplitudes in response to novel stimuli, results revealed a rather different pattern. Simple group comparisons showed that HR adolescents clearly displayed significantly smaller novelty-P300 amplitudes than their NR counterparts. Regression analyses again indicated that externalizing symptoms significantly predicted a smaller novelty-P300 (*p*=.05). However, in contrast to the target-P300, having a parental history of SUD appeared to be the strongest predictor of novelty-P300 amplitude, and risk-group status explained a unique proportion of the variance in novelty-P300 above and beyond all other variables. None of the familial, environmental, physiological or behavioral variables did impact upon novelty-P300 amplitude. Since the novelty-P300 is thought to reflect an orienting response toward unexpected stimuli (i.e., an involuntary shift of attention that is essential for appropriate cognitive processing; e.g., [84], the reduced novelty-P300 in offspring of addicted parents supports the idea of a hyposensitivity of the stimulus-driven attentional system. Although tentatively, our results thus suggest that whereas the target-P300 may merely represent a general vulnerability for a broader spectrum of externalizing problems, novelty-P300 amplitude reductions may point to a more specific risk factor for the development of SUD. 

An intriguing question then remains why this P300 amplitude reduction in response to distracter stimuli is specifically related to risk for SUD. One possibility that may offer an explanation for our findings could be that, rather than reflecting an orienting response, the novelty-P300 responses may be linked to the no-go aspects of our paradigm [85]. Participants were required to inhibit a response when they encountered the distracter stimuli (12% of the time). The P300 amplitude elicited by these rare stimuli in our paradigm may therefore also be a manifestation of response inhibition, thereby acting as a *No-Go* P300 response. Generally, a “No-Go” P300 is elicited in a three-stimulus oddball if *non-novel* repeated stimuli are used as distracters (inserted into the sequence of target and standard stimuli) that do not require a response [85,86]. This type of distracter stimuli generates a P300 with maximum amplitudes over the central/parietal areas (e.g., [87]), and has been related to response inhibition mechanisms (for a review concerning the different P300 components, see Polich [11]). Although our distracter stimuli were abstract, *non-repeating* stimuli, it might be possible that they were perceptually not distinctive enough. This might be evidenced by the characteristics of this component, as our distracter P300 response had a topography that is more equal to the typical No-Go P300 (i.e., a more central/parietal distribution) than to the classical “novel” P300 (i.e., more frontal/central scalp distribution). Hence, the reduced P300 response to infrequent distracter stimuli in our HR sample may also reflect impaired inhibitory control in these adolescents, a cognitive dysfunction that has been frequently linked to (risk for) SUDs (e.g., Ivanov et al. [88]). In this view, vulnerability for externalizing problems might be characterized by reduced deployment of attentional resources (i.e., impaired attentional control), whereas impaired response inhibition may be particularly central to risk for SUD. However, evidence of a reduced “no-go” component indicating impaired inhibitory control in our HR sample was not corroborated by the behavioral data, as HR adolescents did not make more commission errors in response to the novel stimuli and accuracy rates were comparable across groups. Possibly, our paradigm was too easy to make errors and to reveal group differences in performance. Nevertheless, this ‘impaired inhibition hypothesis’ is a tentative hypothesis which we deem worthy of in-depth consideration in future work. The HR adolescents overall responded faster than the NR controls which is typical for impulsive populations [89].

A final note should be made regarding adolescents’ habitual substance use behavior. Though previous studies evidenced that P300 amplitude is sensitive to several drugs of (ab)use [43] and frequency of alcohol and nicotine use in the present study were significantly inversely correlated to the target-P300, results of our regression analyses showed that habitual substance use behavior in our sample did not make significant and unique contributions to the variance in both target- and novelty-P300 amplitudes. This may suggest that the difference between risk groups with respect to the novelty-P300 in our study is not secondary to prolonged heavy exposure to alcohol or other drugs, but may reflect an inborn vulnerability to the development of SUDs.

The present results and conclusions should, however, be interpreted in light of an important caveat. The nature of our study is exclusively quasi-experimental and correlational, and it should be noted that checking for the existence of causal link and examining the degree to which P300 anomalies are vulnerability factors to SUDs or (directly/indirectly) caused by them cannot be ascertained with the present methodology. Some differences between groups, for example, could be accounted by participants’ exposure to substances (and particularly alcohol) during pregnancy and breastfeeding, a factor we could not control for. Moreover, other potential relevant confounding variables such as family functioning, attachment style, and the influence of peers have not been taken into account. Therefore, the conclusions derived from our results must be considered only as conceivable hypotheses until they are confirmed in prospective studies with a longitudinal design.

Taken together, although we explicitly acknowledge the limitations of the study stated above, the present results provide important insights into the determinants of reduced P300 amplitudes in children of addicted parents and their normal-risk counterparts. Parental SUDs were associated with an array of unfavorable offspring outcomes, emphasizing the importance of evaluating familial, environmental and behavioral background variables in at-risk adolescents. The present study demonstrated that overall, the P300 response to both target- and novel stimuli differentiated the HR and NR adolescents. Smaller P300 amplitudes were found in HR offspring, although the effect on target-P300 only approached significance. The effect of having a parental history of SUD on target-P300 vanished when parental rearing, life adversities, stress-reactivity, frequency of substance use and psychopathology were taken into account. Externalizing problem behavior was a strong predictor of the target-P300 amplitude. In contrast, risk group status uniquely predicted novelty-P300 amplitude reductions above and beyond all other variables. Overall, this pattern of results thus underscores the importance of conducting multiple assessments when examining important cognitive processes in at-risk adolescents. The present findings tentatively suggest that the P300 amplitude reduction in response to novel stimuli might be a more specific endophenotype for SUD than the P300 amplitude to task-relevant target stimuli.
